# Reproducibility of a modified posterior reconstruction during robotic intracorporeal neobladder reconfiguration

**DOI:** 10.1590/S1677-5538.IBJU.2022.0417

**Published:** 2022-11-20

**Authors:** Bernardo Rocco, Simone Assumma, Tommaso Calcagnile, Mattia Sangalli, Filippo Turri, Salvatore Micali, Giorgia Gaia, Giorgio Bozzini, Maria Chiara Sighinolfi

**Affiliations:** 1 ASST Santi Paolo e Carlo Department of Urology Milano Italy Department of Urology, ASST Santi Paolo e Carlo, Milano, Italy; 2 University of Modena and Reggio Emilia Department of Urology Italy Department of Urology, University of Modena and Reggio Emilia, Italy; 3 ASST Santi Paolo e Carlo Department of Gynecology and Obstetrics Milano Italy Department of Gynecology and Obstetrics, ASST Santi Paolo e Carlo, Milano, Italy; 4 ASST Lariana Como Italy ASST Lariana, Como, Italy

**Keywords:** Cystectomy, Anastomosis, Surgical

## Abstract

**Objective:**

Robotic intracorporeal neobladder reconstruction is a complex procedure in which the approximation of the reservoir to the urethral stump can be a demanding step.

The aim of the study is to evaluate the reproducibility of a modified posterior reconstruction (PR) during the reconfiguration of intracorporeal neobladder after robot assisted radical cystectomy (RARC).

**Materials and Methods:**

From July 2021 to July 2022, 35 RARC were performed, and 17 patients underwent intracorporeal neobladder reconstruction. A PR was planned in males (14). Intra- and peri-operative data were collected.

Surgical technique: RARC and node dissection are performed. Afterwards, 40-cm ileal segment is isolated; the portion with the more adequate mesenteric length is brought down to the pelvis. A modified PR is performed with a double-armed barbed suture: a first layer connects the Denonvillier's fascia to the rhabdosphincter in a running fashion; the second layer is created with the other arm and approximates the posterior side of the ileal segment towards the urethral stump. In the anterior caudal part of the ileum, a 1.5-cm incision is made to realize the neobladder neck; the neovesical-urethral anastomosis is performed with a second bidirectional suture.

**Results:**

Anastomotic and PR time were 14 (range 7-20) and 5 minutes (4-8), respectively. A single Clavien IIIa complication was recorded in a patient who underwent NAC and had a *C. albicans* superinfection in the post-operative course. All patients were discharged with complete or acceptable bladder voiding. Twelve patients with follow-up >90-days reported a satisfying daytime continence.

**Conclusions:**

PR represents a simple technical refinement that improves neobladder-urethral anastomosis by favoring ileal approximation to the urethral stump and decreasing anastomotic tension.

## INTRODUCTION

Radical cystectomy (RC) is the mainstay of treatment of bladder cancer (1, 2). Even if bladder sparing strategies are challenging the role of surgery (1, 3), technical and technological advances are making the surgical procedure less invasive. RC is a complex procedure that may involve the combined surgery of the urinary and gastrointestinal tract (4–6). It can be performed either open, laparoscopically and robotically; current EAU Guidelines suggest that no approach is over another. Robot-assisted radical cystectomy (RARC) with intracorporeal neobladder have been recently recognized as an approach able to preserve global health related quality of life (7, 8); however, it has been the slowest adopted since challenging and time-consuming (9). To simplify the reconstruction of reservoir, several technical refinements have been described (9–12). One of the most challenging parts of robotic intracorporeal neobladder reconstruction is the approximation of the ileal segment to the urethral stump. Some tricks were developed to this purpose, such as decreasing the Trendelenburg or the use of vessel loops around the bowel to maximize intestinal brought down (13).

A modified posterior reconstruction (PR) was proposed as well, to facilitate neobladder-urethral anastomosis and enhance urinary continence recovery (13). The technique has been used with a modified Studer neobladder (13): the double-layer suture approximates the Denonvillier's fascia to the rhabdosphincter and then connects the posterior site of neobladder neck to urethral stump before the anastomosis.

The current paper aims to determine whether the approach is easily reproducible with other reconfigurations; to this purpose, a PR during a modified Y-shaped neobladder reconstruction was evaluated in male patients.

## MATERIALS AND METHODS

Between July 2021 and July 2022, 35 patients underwent RARC for high-grade and/or muscle-invasive bladder cancer. The study aims to evaluate the feasibility and reproducibility of a PR in the setting of neobladder reconstruction; inclusion criteria were patients aged 18-80, male gender, undergoing orthotopic diversion. Outcome measures were anastomotic and PR time; intra- and post-operative complications were recorded as well. The removal of the trans-urethral catheter was planned between PO Day 12 and 16. All procedures were performed by a single surgeon (BR) highly expert in robotic pelvic and reconstructive surgery. Follow up was recorded at 30-days, 3, 6, 12 months.

## SURGICAL TECHNIQUE

A full video of the technique is available at https://youtu.be/PyZ8nGOmd2Y.

An open supra-umbilical access is performed with the placement of an Alexis device. The other robotic ports and those for the assistant are created according to the Asimakipoulos and Gaston description (14). The procedure starts with the identification and isolation of ureters bilaterally from above iliac vessels until bladder insertion. At bladder level the ureter is closed with median size Hem-o-lok and then sectioned. In males, the peritoneum at seminal vesicle level is incised and the plane between Denonvilliers’ fascia and the posterior face of the prostate is developed (between bladder and vagina in females). Lateral aspects of the bladder are developed bilaterally, and vesical pedicles are clipped and transected. Inverse U peritonectomy is carried out between the two internal inguinal rings, umbilical arteries are transected and access to the Retzius space is created. In males, the preservation of neurovascular bundle is performed when recommended, with a high anterior release of the peri-prostatic nerves. The anterior aspect of the bladder is developed, and the Santorini complex is severed and then sutured. The urethra is isolated and incised after a large hem-o-lok is placed to prevent urine spillage. The urethral stump is maintained as long as possible. Frozen sections of distal ureters and urethra are performed, meanwhile, an extended pelvic nodal dissection is carried out bilaterally.

**Figure 1:**
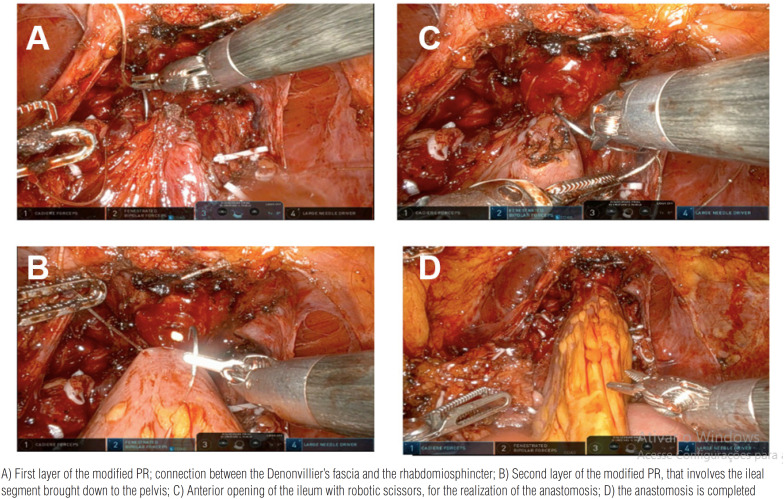
Intra-operative images of PR

**Figure 2:**
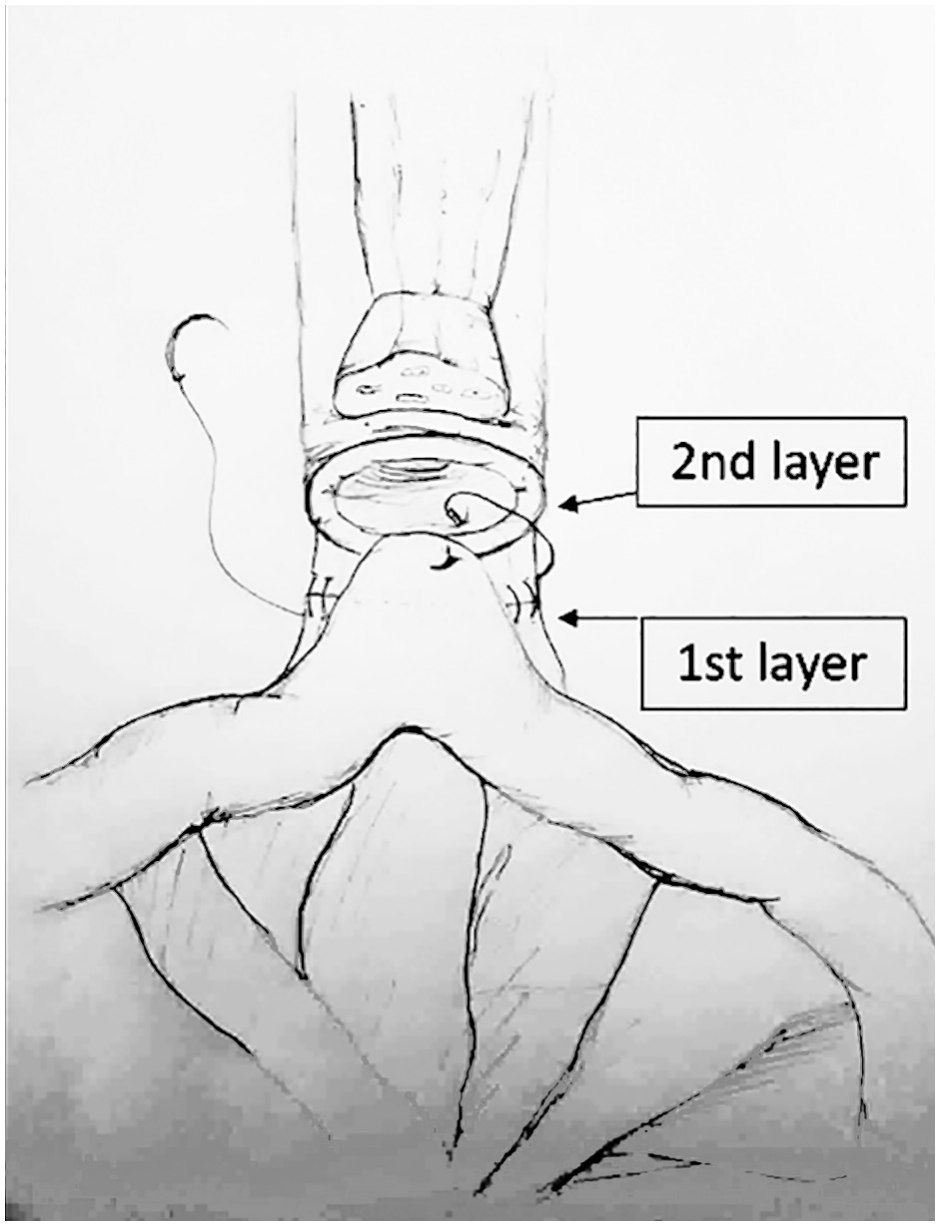
Drawing depicting the two layers of posterior reconstruction during intra-corporeal neobladder reconstruction. Layer 1: the more posterior, connection between the Denonvillier's fascia and the posterior rhabdomiosphincter; Layer 2: connection between the ileum and the urethral stump

Afterwards, a 40-50 cm ileal segment is isolated; the portion with the more adequate mesenteric length is chosen to be brought down to the pelvis. The reconfiguration of the neobladder starts from the posterior reconstruction and vesico-urethral anastomosis. The median part of the isolated ileal segment is pushed towards the urethral stump. A modified PR is performed with a double-armed barbed suture (Stratafix3–0, Ethicon™) used as a running suture. The first layer connects the Denonvillier's fascia and the rhabdosphincter (Figure-1A), whereas the second layer approximates the posterior side of the ileal segment towards the urethral stump, by using the other side of the double armed suture (Figure-1B). While tying the suture, caudal approximation of the ileum is supported by the assistant and by the fourth arm. In the anterior caudal part of the ileal loop, a 1.5-cm incision is made with robotic scissor to realize the neobladder neck; the neovesical-urethral anastomosis is performed with a second 3-0 Stratafix bidirectional needle (Figure-1C). Figure-1D depicts the final view of the ileo-urethral anastomosis. A schematic drawing is provided in Figure-2.

The isolation of the segment is made cranially on each side by using a mechanical laparoscopic stapler; ileal-ileal anastomosis is performed thereafter. The reverse tubular U-segment of the ileum is configured, and the ileum is detubularized. The reconfiguration of the neobladder starts from the suture of the posterior plane with a 3/0 running barbed suture (3-0 barbed suture); the cranial part is folded downwards and anastomized with the bladder neck. The lateral horns of the reservoir are closed in their anterior aspect with a 3-0 running suture. The neobladder it tested for leakage; then uretero-neobladder anastomosis is performed with a direct anastomosis of each spatulated ureter in the dorsal part of the horns (4-0 polydioxanone). Ureteral stents are placed before suturing the anterior plate and are brought out through the anterior abdominal wall.

## RESULTS

Seventeen out of 35 patients underwent an intracorporeal neobladder reconfiguration. The series consisted of 14 males and 3 females; the latter were pre-operatively counseled with the gynecologist for a sexual sparing approach. All male patients received a PR associated to the neobladder-urethral anastomosis. A detailed description of patients who underwent neobladder reconstruction with PR is provided in Table-1.

**Table 1:** Full descriptive analysis of demographics, clinical and pathological data of patients undergoing neobladder reconstruction.

Age	Mean ? SD (range)	64.5 years ? 7.5 (48-75) years
**Gender**	Female (%)	3 (17.6%)
	Male (%)	14 (82.4%)
**BMI**	Mean ? SD (range)	27, ? 1.4 (22.1-31.7)
**Smoker**	Yes (%)	7 (41.2%)
	No (%)	6 (35.3%)
	Previous (within 5 years)	4 (23.5%)
**Histological examination (after TURBT)**	T1 high/very high risk (%)	5 (29.4%)
	T2 (%)	9 (53%)
	Recurrent/multifocal CIS (%)	3 (17.6%)
**Previous BCG therapy**	Yes (%)	4 (23.5%)
	No (%)	13 (76.5%)
**Neoadjuvant CHT**	Yes (%)	4 (23.5%)
	No (%)	13 (76.5%)
**Anastomotic time**	Mean (range)	14 (7-20) minutes
**PR time**	Mean (range)	5 (4-8) minutes
**Intra-operative complications**	Yes (%)	0 (0%)
	No (%)	17 (100%)
**Post-operative complications**	Yes (%)	3 (17.6%)
	No (%)	14 (82.4%)
**30-days readmission**	Yes (%)	1 (5.9%)
	No (%)	16 (94.1%)
**Histologic stage: T (tumor)**	T0	3 (17.6%)
	Ta	0 (0%)
	Tis	3 (17.6%)
	T1	1 (5.9%)
	T2	6 (35.3%)
	T3	2 (11.8%)
	T4	2 (11.8%)
**Histologic stage: N (lymph nodes)**	N0	12 (70.6%)
	N1	4 (23.5%)
	N2	1 (5.9%)
	N3	0 (0%)
**Histologic grade**	LG	3 (17.6%)
	HG	14 (82.4%)
**Histological stage: R (surgical margins)**	R0	17 (100%)
	R+	0 (0%)

Anastomotic and PR time were 14 (range 7-20) and 5 minutes (4–8), respectively. No intra-operative complications were recorded. Post-operative course was uneventful in all patients except two cases of neobladder leakage (one of them requiring nephrostomy tube placement) and a single case of persistent hematuria due to inadvertent catheter dislodgement. The patient who underwent nephrostomy drainage had received a prior neoadjuvant chemotherapy and was affected by a*C. albicans* urinary superinfection. In the absence of leakage, the neobladder-urethral catheter was removed within PO Day 12 and 16. All patients were discharged with ultrasonographic confirmation of complete or acceptable bladder voiding (< 50-100 mL). A single patient was re-admitted within 30 days due to febrile UTI. Currently, 12 male patients have > 90-day follow up (range 3-12 months); all of them report a satisfactory daytime continence (no pad use for 10 patients and 0/1 pad/day for 2 patients) and a mild degree of a nighttime incontinence (use of 1 pad/night).

## DISCUSSION

A posterior reconstruction with the involvement of the ileal loop within the second layer is an easy and reproducible step of robotic intracorporeal neobladder reconstruction.

Recently, Checcucci et al. described a RARC series with a simple posterior reconstruction, which includes the Denonvillier's fascia and a peritoneal flap from the Douglas pouch: unlike our procedure, the technique fails to comprise the ileal loop within the reconstruction (15).

A modified PR incorporating the ileal segment has been previously described in a series of robotic Studer neobladder performed at the Karolinska University Hospital (13). Authors found that the technique could be easily performed intracorporeally with a negligible additional console time; furthermore, a 100% and 44% daytime and nighttime continence at 12-months were recorded, though the small series and the absence of a control group precluded any conclusion to this point.

A posterior reconstruction has been long used during radical prostatectomy. Since its first description in 2006 (16), the technique gradually spread and has been successfully adapted to minimally invasive surgery. Its benefit on early urinary continence recovery after radical prostatectomy has been reported in a Cochrane review by Rosemberg et al. (17) and another meta-analysis (18).

Actually, after the disruption occurring during pelvic surgery, PR restores the anatomical and functional length of the rhabdosphincter and reestablish the continuity of the musculofascial plate. Beyond the use for continence recovery, PR may represent a support for anastomosis, by reducing the tension and improving the approximation between the bladder neck and urethral stump.

The same advantages apply to the neobladder-urethral anastomosis after RARC.

Unlike open surgery, the first step of most robotic intracorporeal neobladder is the ileo-urethral anastomosis performed prior to the detubularization and reconfiguration of the ileum itself. Some tricks enable the approximation of the ileal segment to the pelvis, such as the use of two vessel loops passed around the intestine through the mesentery to facilitate the traction toward the urethra. However, the ileum wall has a soft tissue texture that can be damaged by instruments tractions, especially in case of inadvertent conflicts, or by the tension itself. A modified posterior reconstruction prior to the anastomosis may facilitate the approximation of the ileum toward the urethral stump and reduce the tension on the bowel wall. The posterior support to the anastomosis provides advantages also for catheter placement, especially in cases of difficult unaligned bladder neck.

The modified PR appears not to impair operative time. Overall, the procedure takes approximately 6 minutes, similar to the length reported in the series with Studer diversion.

The limitation of the current study is the small sample size and the absence of a control group. Thus, any consideration about urinary continence is precluded. Nevertheless, the primary endpoint of the study was to test the *reproducibility* of the PR rather than to prove its effectiveness to improve continence recovery.

In conclusion, a PR that involves the Denonvillier's fascia, the posterior site of neobladder neck and the rhabdosphincter is a simple and reproducible step that may maximize the approximation of the reservoir toward the pelvis, reduce anastomotic tension and simplify robotic intracorporeal reconstruction of orthotopic neobladder.
